# Impact of administering cardiac medication to small-breed dogs with preclinical myxomatous mitral valve disease on survival after congestive heart failure onset

**DOI:** 10.1080/01652176.2025.2560412

**Published:** 2025-09-21

**Authors:** Sin-Wook Park, Keon Kim, Yoon-Jung Do, Jong-Won Lee, Woong-Bin Ro, Chang-Min Lee

**Affiliations:** a Department of Veterinary Internal Medicine, College of Veterinary Medicine and BK21 FOUR program, Chonnam National University, Gwangju, Republic of Korea; b Re-born Animal Medical Center, Busan, Republic of Korea; c Division of Animal Diseases & Health, National Institute of Animal Science, Rural Development Administration, Wanju-gun, Republic of Korea

**Keywords:** Canine, cardiogenic pulmonary edema, Chronic mitral valve disease, pimobendan

## Abstract

Myxomatous mitral valve disease (MMVD) is the most common cardiovascular disease in small-breed dogs, and some affected dogs develop congestive heart failure (CHF). Although pimobendan is recommended to delay the onset of CHF, its effect on survival following CHF onset development remains unclear. This retrospective study evaluated the survival prognosis of 143 small-breed dogs diagnosed with first-time CHF due to MMVD, comparing pretreated (*n* = 54) and untreated (*n* = 89) groups. Pretreated dogs received cardiac medications including pimobendan for at least five weeks before CHF onset. Pretreated dogs had a significantly larger normalized left ventricular internal diameter (LVIDDN; *p* = 0.002) and higher left atrium-to-aortic root ratio (LA/Ao; *p* = 0.044) at CHF onset than untreated dogs. The median survival time after CHF onset was significantly longer in untreated dogs (481 days, 95% confidence interval (CI) 393–569 days) than in pretreated dogs (212 days, 95% CI 73–351 days; *p* = 0.028). Univariable Cox proportional hazards analysis identified pretreatment (*p* = 0.031), chordae tendineae rupture (*p* = 0.011), and the LA/Ao (*p* < 0.001) as significant predictors of survival. Our findings suggest that the administration of cardiac medications, including pimobendan, prior to the onset of CHF was not independently associated with improved survival following CHF.

## Introduction

Myxomatous mitral valve disease (MMVD) is the most common cardiovascular disease in small-breed dogs, accounting for approximately 70% of cases of heart disease (Kim et al. 2017). The progression of MMVD results in congestive heart failure (CHF), which may eventually cause dyspnea, syncope, cyanosis, and even death (Kim et al. 2017; Mizuno et al. 2017). Approximately 30% of patients with MMVD are reported to develop CHF, with a median survival times (MST) of 136–367 days following the onset of CHF (Häggström et al. 2008; Borgarelli and Haggstrom 2010; Kim et al. 2017; Mizuno et al. 2017).

Pimobendan is a phosphodiesterase inhibitor that enhances myocardial contractility and induces vasodilation without requiring increased energy (Kanno et al. 2007). Previous studies have reported that the administration of pimobendan in dogs with preclinical MMVD with evidence of cardiomegaly may delay the onset of CHF and reduce heart size (Boswood et al. 2016; 2018; Keene et al. 2019). In addition, one study demonstrated that the concurrent use of pimobendan and diuretics upon CHF onset extends survival time compared with diuretic monotherapy (Häggström et al. 2008). Accordingly, the American College of Veterinary Internal Medicine (ACVIM) consensus guidelines for the diagnosis and treatment of chronic valvular heart disease in dogs recommend the use of pimobendan for the chronic management of MMVD in dogs with stage B2 (preclinical), C, and D disease (Keene et al. 2019; Wess et al. 2020).

To the best of our knowledge, no studies have yet compared the survival time following the onset of CHF between dogs that were preemptively administered cardiac medications including pimobendan and those that received no medication prior to CHF onset but were subsequently treated with similar therapeutic protocols after CHF diagnosis, including pimobendan. Therefore, we aimed to retrospectively assess whether the preemptive use of cardiac medications influences the survival prognosis following CHF development in small-breed dogs with MMVD. Furthermore, we aimed to evaluate whether there are differences in echocardiographic parameters at the time of CHF onset based on preemptive use.

## Materials and methods

### Animals

Medical records from the Re-born Animal Referral Medical Center collected between April 2021 and February 2025 were retrospectively reviewed to identify small-breed dogs diagnosed with CHF associated with MMVD. The collected data included age, breed, sex, body weight, underlying medical conditions, physical examination results, clinical pathological data, diagnostic imaging findings, and treatments administered.

The inclusion criteria were as follows: dogs aged ≥6 years with a body weight ≤10 kg that were diagnosed with first-onset CHF consistent with ACVIM stage C during hospitalization at our facility. CHF was defined as the presence of clinical signs of left-sided CHF (e.g. cough, tachypnea, respiratory distress), supported by thoracic radiographic evidence of pulmonary edema, and confirmed within 24 h of presentation. In addition, echocardiographic evidence of MMVD—characterized by thickened mitral valve leaflets with or without chordae tendineae rupture (CTR), along with left atrial (LA) and left ventricular (LV) enlargement—was required to confirm underlying MMVD as the etiology. The onset of CHF was defined as the date on which pulmonary edema was first radiographically confirmed, in conjunction with compatible clinical signs and echocardiographic findings. Dogs were enrolled only if this was their first documented CHF episode.

Dogs were excluded if they had congenital cardiac disease, other acquired cardiac conditions (e.g. bacterial endocarditis, dilated cardiomyopathy, pulmonary stenosis), or were on cardiac medications other than pimobendan, angiotensin-converting enzyme inhibitors (ACEIs), spironolactone, sildenafil, or amlodipine. Dogs with an unidentified medication history prior to hospitalization, those with previous CHF episodes, or those that died without responding to initial CHF treatment within the first hospitalization were also excluded.

Dogs were divided into two groups: the pretreated group, which consisted of dogs that received cardiac medications (including pimobendan) for at least five weeks prior to CHF onset, and the untreated group, which included dogs that did not receive any cardiac medication prior to CHF diagnosis. All pretreated dogs received cardiac medications only after being classified as stage B2 MMVD according to ACVIM guidelines, based on physical and imaging evidence of both LA and LV enlargement. After hospital discharge, dogs were treated according to the ACVIM consensus guidelines for the management of dogs with stage C MMVD, and dose and intervals were subsequently adjusted according to individual clinical evaluations (Keene et al. 2019).

### Echocardiographic data

All echocardiographic examinations were performed by two board-certified veterinary radiologists and reviewed by one board-certified veterinary internist. The value recorded for each measurement consisted of the average of three cardiac cycles. The left-atrium-to-aortic root ratio (LA/Ao) was obtained from the right parasternal short-axis two-dimensional (2D) view as previously described, and the left ventricular internal diameter at end-diastole (LVIDd) and left ventricular internal diameter at end-systole (LVIDs) measured on the M-mode echocardiogram were obtained from the right parasternal short-axis view (Boswood et al. 2018). M-mode values were used to derive the fractional shortening percentage (FS%). Normalized dimensions were calculated according to the following formula: normalized LVIDd (LVIDDN) = LVIDd(cm)/(BW (kg))^0.294^ (Boswood et al. 2018). CTR diagnosis was based on detection of a mitral valve flail with the leaflet tip pointing back into the left atrium during systole, confirmed in 2D imaging planes using the left and right parasternal 4-chamber views.

From a left apical 4-chamber view, transmitral Doppler indices including peak velocities of the early diastolic (E) and late diastolic (A) waves and the E to A ratio (E/A) were recorded. The maximal flow velocity of the tricuspid regurgitation (TR) jet from the following three different views was assessed: left parasternal (LPS) apical 4-chamber view, LPS long-axis view of the right auricle, and LPS cranial transverse view of the tricuspid valve. This measurement was then applied to estimate the systolic pressure gradient across the tricuspid valve, representing the systolic pulmonary artery pressure (PAP). A diagnosis of pulmonary hypertension (PH) was made when the peak TR jet velocity exceeded 3.4 m/s. In cases in which the TR flow was absent, the maximal PR jet velocity was used to identify PH, and its measurement was applied to estimate the mean PAP, with a peak PR jet velocity ≥2.2 m/s indicating PH (Channgam et al., 2024).

### Statistical analysis

All statistical data were analyzed using commercial software (IBM SPSS Statistics, version 27, IBM Co., United States). Data were tested for normality using the Shapiro-Wilk test. Descriptive statistics are reported as the mean ± standard deviation (SD) for normally distributed continuous variables and as the median (interquartile range, IQR) for non-normally distributed continuous variables. To compare baseline variables between the pretreated and untreated groups depending on the distribution of the variables, the Chi-square test or Fisher’s exact test was used for categorical variables and the independent samples t-test or Mann-Whitney U-test was used for continuous variables.

Dogs were censored at the time of last follow-up if they were alive at the study conclusion, lost to follow-up, or died due to non-cardiac causes. Univariable Cox proportional hazards analysis (age, sex, body weight, creatinine, LVIDDN, FS, LA/Ao, E peak, E/A ratio, PH, CTR, pretreatment) was performed to identify potential predictors of mortality by calculating hazard ratios (HRs) with 95% confidence intervals (CIs). The significant factors (pretreatment, CTR, LA/Ao) from the univariable Cox proportional hazard analysis (*p* < 0.1) were included in the multivariable Cox proportional hazards analysis. Kaplan-Meier survival curves with log-rank tests were employed to compare median survival times between pretreated and untreated groups. Survival time was calculated from the date of each dog’s first diagnosis of CHF. Statistical significance was set at *p* < 0.05.

## Results

### Characteristics

The baseline characteristics of the general population and of pretreated and untreated dogs are summarized in Table 1, and all data were measured at the onset of the first CHF episode. A total of 143 client-owned dogs were enrolled in the study. The most represented breeds were Maltese (*n* = 75), mixed breed (Borgarelli et al. 2015), Shih Tzu (Baron Toaldo et al. 2018), Pomeranian (Beaumier et al. 2018), Poodle (Wess et al. 2020), and Chihuahua (Kanno et al. 2007), and an additional seven breeds were represented by two or fewer dogs each. A total of 70 were male (49.0%), and 64 of these were neutered; 73 dogs were female (51.0%), and 60 of these were spayed. Age, sex, body weight, heart rate, systolic arterial blood pressure, creatinine level, FS%, E max, E/A ratio, and presence/absence of PH were not significantly different between pretreated and untreated dogs. CTR was identified in 72 of the 143 dogs (50%) and was more common in untreated dogs with stage C MMVD (*p* < 0.001). A ruptured chordae tendineae had been attached to the anterior mitral valve leaflet in all dogs, and one dog had rupture of the chordae tendineae anchored to both the anterior and posterior leaflets. Untreated dogs had significantly smaller LVIDDN and LA/Ao values than treated dogs. Dogs with CTR had significantly smaller LVIDDN (dogs with CTR: 1.84 ± 0.32 cm, dogs without CTR: 1.95 ± 0.25 cm; *p* = 0.019) and LA/Ao values (dogs with CTR: 1.94 ± 0.33, dogs without CTR: 2.22 [2.00–2.58]; *p* < 0.001) than those without CTR.

**Table 1.** Descriptive statistics for all 143 dogs with congestive heart failure, untreated dogs, and pretreated dogs.

Variable	All (*n* = 143)	Untreated group (*n* = 89)	Pretreated group (*n* = 54)	*P*-value
Age (years)	11.9 ± 2.7	11.8 ± 2.7	12.2 ± 2.7	0.365
Sex (male/female)	70/73 (49%/51%)	41/48 (46%/54%)	29/25 (54%/46%)	0.376
Body weight	3.8 (2.9–4.8)	3.9 (2.9–4.8)	3.5 (2.8-5)	0.921
Heart rate (beats per minute)	160 (144–180)	156 (144–180)	170 (160–180)	0.801
Systolic arterial blood pressure (mmHg)	125 ± 32	125 ± 35	126 ± 28	0.864
Creatinine (mg/dL)	0.9 (0.7–1.2)	0.9 (0.7–1.2)	0.9 (0.8–1.3)	0.106
LVIDDN	1.9 ± 0.3	1.8 ± 0.3	2.0 ± 0.3	0.002
FS (%)	58 ± 8	58 ± 8	58 ± 8	0.948
LA/Ao	2.14 ± 0.46	2.08 ± 0.46	2.24 ± 0.45	0.044
E peak	132 ± 30	130 ± 29	136 ± 31	0.307
E/A ratio	1.7 ± 0.8	1.8 ± 0.9	1.6 ± 0.7	0.115
PH (yes/no) (%)	25 (17%)	15 (17%)	10 (19%)	0.856
CTR (yes/no) (%)	72 (50%)	56 (63%)	16 (30%)	<0.001

*P*-value refers to differences between pretreated and untreated dogs. CTR, chordae tendineae rupture; E peak, peak velocity of E wave of transmitral flow; E/A ratio, E-peak velocity to A-peak velocity of transmitral flow; FS, Fractional shortening; LA/Ao, left atrium-to-aortic root ratio; LVIDDN, normalized left ventricular end-diastolic internal diameter indexed; PH, pulmonary hypertension.

### Treatments

Five dogs in the untreated group had been diagnosed with MMVD before experiencing CHF. At baseline, pretreated dogs (*n* = 54) were prescribed cardiac medications before the onset of their first CHF episode, and the median duration of cardiac medication administration was 9 months (3.5–21 months). These dogs received one or a combination of the following drugs: pimobendan (54/54, 100%), enalapril (33/54, 61.1%), benazepril (11/54, 20.4%), spironolactone (40/54, 74.1%), sildenafil (10/54, 18.5%), and amlodipine (2/54, 3.7%); no dog received both ACEIs concurrently. The median (range) doses of each drug administered prior to CHF onset were as follows: pimobendan, 0.25 mg/kg BID (0.25–0.3); enalapril, 0.5 mg/kg BID (0.25–0.5); benazepril, 0.25 mg/kg BID (0.25–0.5); spironolactone, 1 mg/kg BID (0.5–1); sildenafil, 0.5 mg/kg BID (0.5–1.5); and amlodipine, 0.15 mg/kg SID (0.1–0.2).

On discharge, all dogs received a combination of the following drugs: pimobendan (143/143, 100%), furosemide (143/143, 100%), enalapril (80/143, 55.9%), benazepril (23/143, 16.1%), spironolactone (97/143, 67.8%), sildenafil (22/143, 15.4%), and amlodipine (4/143, 2.8%); again, no dog received both ACEIs concurrently. The median (range) doses of each drug administered after CHF onset were: pimobendan, 0.3 mg/kg BID (0.25–0.4); enalapril, 0.5 mg/kg BID (0.25–0.5); benazepril, 0.25 mg/kg BID (0.25–0.5); spironolactone, 1 mg/kg BID (1–1.5); sildenafil, 1 mg/kg BID (0.5–2); and amlodipine, 0.1 mg/kg SID (0.1–0.2).

### Survival analysis

Of the 143 dogs, 75 died or were euthanized during the study period for reasons related to heart disease, 3 dogs died from noncardiac-related causes, and 22 other dogs still were alive at the end of the study. The results of the univariable Cox proportional hazards analysis are shown in Table 2. Three variables were significantly associated with survival and were therefore included in the final multivariable model: pretreatment (yes vs no; *p* = 0.031), CTR (yes vs no; *p* = 0.011), and the LA/Ao (*p* < 0.001). In the final multivariable analysis, only the LA/Ao remained independently associated with survival over the follow-up period (HR: 2.045, 95% CI: 1.286–3.251; *p* = 0.003). The median survival time in the overall population of dogs (*n* = 143) with stage C MMVD was 390 days (95% CI: 284–496). The median survival time was significantly longer in the untreated group (481 days [95% CI: 393–569]) than in the pretreated group (212 days [95% CI: 73–351]; *p* = 0.028). Similarly, dogs with CTR had a significantly longer median survival time (510 days [95% CI: 351–669]) than those without CTR (285 days [95% CI: 134–436]; *p* = 0.009).

**Table 2.** Univariate cox proportional hazards regression associated with outcome in 143 dogs with congestive heart failure.

Variable	Hazard ratio	95.0% confidence interval of the hazard ratio		*P*-value
Age (years)	1.004	0.996	1.013	0.307
Sex	1.074	0.681	1.695	0.758
Body weight	0.965	0.813	1.145	0.680
Creatinine (mg/dL)	1.489	0.921	2.046	0.104
LVIDDN	0.966	0.440	2.122	0.932
FS (%)	1.013	0.981	1.045	0.432
LA/Ao	2.176	1.371	3.454	<0.001
E peak	0.999	0.991	1.006	0.696
E/A ratio	0.825	0.619	1.110	0.191
Pulmonary hypertension	1.218	0.635	2.337	0.552
CTR	0.547	0.344	0.870	0.011
Pretreatment	1.654	1.048	2.612	0.031

## Discussion

In this retrospective study, we evaluated the impact of administering cardiac medication to small-breed dogs with MMVD on survival time after the onset of CHF. MMVD is the most common cardiac disease in dogs, with progression to CHF significantly contributing to morbidity and mortality (Boswood et al. 2016). In clinical practice, some dogs receive treatment before CHF develops, whereas others are diagnosed with MMVD at the time of CHF onset. Understanding the effect of prior cardiac medication use on post-CHF prognosis may provide useful context for prognostic assessment. We confirmed that pretreatment, CTR, and the LA/Ao were associated with survival time, but the multivariable analysis identified the LA/Ao as the only independent predictor of survival. Thus, these results suggest that preemptive therapy in the preclinical stage was not independently associated with improved survival once CHF had developed. Instead, LA enlargement at the time of CHF diagnosis emerged as the key prognostic factor in our cohort.

Our findings indicate that the LA/Ao was a significant prognostic factor for survival. Dogs with a higher LA/Ao at the time of CHF onset had significantly shorter survival times, consistent with the findings of previous studies emphasizing that LA enlargement is an essential indicator of disease severity and progression in dogs with MMVD (Nakamura et al. 2014; Beaumier et al. 2018). LA enlargement reflects chronic volume overload caused by severe mitral regurgitation, leading to myocardial remodeling, increased filling pressures, and impaired cardiac function (Nakamura et al. 2014; Baron Toaldo et al. 2018; Vezzosi et al. 2021). Consequently, elevated LA/Ao values are often associated with advanced cardiac remodeling and limited compensatory capacity, resulting in poorer prognoses once CHF develops (Borgarelli and Haggstrom 2010; Keene et al. 2019). This finding suggests that monitoring left atrial size over time may aid in prognostic assessment and help identify dogs at increased risk of poorer outcomes (Sargent et al. 2015).

The multivariable analysis identified the LA/Ao as the only independent predictor of survival, underscoring its prognostic importance in dogs with CHF secondary to MMVD. Interestingly, dogs in the pretreated group exhibited a significantly greater LA/Ao at CHF onset than untreated dogs. Furthermore, dogs with CTR showed a significantly lower LA/Ao than those without CTR. These findings suggest that preemptive medication administration might delay CHF onset until more advanced LA remodeling occurs, resulting in higher LA/Ao values at the time of CHF diagnosis. In contrast, dogs experiencing CTR likely developed CHF acutely, potentially before significant LA enlargement could occur. Such differences in disease progression and cardiac remodeling may explain why the LA/Ao remains an essential independent predictor and provides superior prognostic information over pretreatment status or the presence of CTR. Therefore, evaluating the LA/Ao at CHF onset should be emphasized as a critical aspect of clinical management and prognostication in dogs with MMVD.

In this study, CTR was identified in 50% of dogs with CHF, which is higher than previously reported (Serres et al. 2007; Sargent et al. 2015). This discrepancy may have resulted from referral bias or differences in breed composition. Notably, CTR occurred more frequently in untreated dogs, suggesting acute disease progression leading to sudden CHF onset before medication could be initiated. This is supported by the smaller echocardiographic parameters (LVIDDN, LA/Ao) observed in dogs with CTR than in dogs without CTR.

In dogs with CHF, the presence of CTR was unexpectedly associated with prolonged survival compared to those without CTR. This finding contrasts with the conventional understanding that CTR reflects more advanced MMVD, which is typically associated with severe mitral regurgitation and a poorer prognosis (Ettinger and Buergelt 1969; Sargent et al. 2015). One potential explanation for this finding is that CTR may precipitate the onset of CHF before extensive structural remodeling of the left ventricular myocardium has occurred, leaving more compensatory capacity at the time of decompensation (Kim et al. 2014). This relatively acute event could help explain the longer survival once CHF was diagnosed in these dogs. Nevertheless, these findings should not be interpreted as indicating that the presence of CTR confers a better prognosis in dogs with MMVD because when asymptomatic dogs with CTR are considered, they may actually have a poorer prognosis (Serres et al. 2007; Sargent et al. 2015; Boswood et al. 2016).

Pimobendan has been shown to delay the onset of CHF in dogs with preclinical MMVD through its positive inotropic and vasodilatory effects, which reduce cardiac workload and slow cardiac remodeling (Boswood et al. 2016; 2018; Klein et al. 2021). However, in this study, the administration of pimobendan in dogs with preclinical MMVD did not result in a significant survival advantage following CHF onset. One possible explanation is that the structural and functional benefits from pimobendan during the preclinical stage may have already reached their limit by the time CHF developed. In addition, dogs with preclinical disease receiving pimobendan might have experienced slower disease progression initially, allowing for greater compensatory remodeling; thus, CHF could have occurred at a more advanced stage of myocardial deterioration than in untreated dogs (Boswood et al. 2018; Klein et al. 2021). Likewise, dogs that received preemptive pimobendan may have developed CHF at a more advanced disease state than untreated dogs, as pretreated dogs showed a larger LVIDDN and a higher LA/Ao than untreated dogs. Consequently, any survival benefit conferred in the preclinical phase may no longer be apparent once CHF is present.

PH was identified in 17% of our study population. This prevalence is lower than previously reported values (33–39%) in dogs with MMVD, which may be due to the use of a more stringent TR velocity cutoff (≥3.4 m/s) for PH diagnosis in this study (Serres et al. 2007; Borgarelli et al. 2015). Unlike earlier studies that suggested PH as a negative prognostic factor, the presence of PH did not significantly affect survival time in our cohort (Borgarelli et al. 2015). This discrepancy may be related to the fact that our study focused exclusively on dogs with ACVIM stage C MMVD, in which other factors such as the severity of left-sided heart failure and therapeutic response may have a greater impact on survival (Beaumier et al. 2018).

This study has several limitations that should be considered. First, the retrospective nature of the study introduces potential biases, such as variations in treatment protocols, owner compliance, and follow-up duration. Second, because follow-up echocardiographic examinations were not available for all dogs, it was not possible to assess changes in echocardiographic parameters. Third, PH was diagnosed based on the peak velocity of TR or PR; the severity of PH was not evaluated, and other potential causes of PH apart from MMVD could not be ruled out. Therefore, the prognostic impact of PH severity could not be evaluated, potentially influencing the survival analysis. Furthermore, the majority of pretreated dogs received multiple cardiac medications concurrently, which limits our ability to isolate the specific contribution of pimobendan to the observed outcomes. Further studies are necessary to clearly delineate individual drug effects. Despite these limitations, this study provides valuable insights into the impact of administering cardiac medication to dogs with preclinical MMVD on survival outcomes after CHF onset.

In conclusion, our results indicate that an increased LA/Ao at the time of CHF diagnosis was the only independent predictor of post-CHF survival in dogs with MMVD. While pretreatment and CTR were associated with survival time in univariable analyses, their significance did not persist in the multivariable model, suggesting that baseline cardiac remodeling, rather than pretreatment status, may have been the primary driver of survival differences. Accordingly, echocardiographic assessment of LA/Ao may aid in prognostic evaluation and disease monitoring in clinical practice. Further investigations are warranted to refine therapeutic guidelines and enhance outcomes for dogs with MMVD.

## List of abbreviations

ACVIM: American College of Veterinary Internal Medicine
A: Late diastolic wave
ACEI: Angiotensin-converting enzyme inhibitor
CHF: Congestive heart failure
CI: Confidence interval
CTR: Chordae tendineae rupture
E: Early diastolic wave
E/A: E-wave to A-wave ratio
FS%: Fractional shortening percentage
HR: Hazard ratio
IQR: Interquartile range
LA: Left atrial
LA/Ao: Left atrium-to-aortic root ratio
LPS: Left parasternal
LV: Left ventricular
LVIDD: Left ventricular internal diameter at end-diastole
LVIDDN: Normalized left ventricular internal diameter at end-diastole
LVIDs: Left ventricular internal diameter at end-systole
MMVD: Myxomatous mitral valve disease
MST: Median survival time
PAP: Pulmonary artery pressure
PH: Pulmonary hypertension
PR: Pulmonic regurgitation
SD: Standard deviation
TR: Tricuspid regurgitation
